# Sexual health behaviours and partner notification practices related to sexually transmitted infections in young adults in Ireland

**DOI:** 10.1007/s11845-024-03668-8

**Published:** 2024-03-22

**Authors:** Finiki Nearchou, Clodagh Flinn, Diarmuid Mc Laughlin, Rachel Niland

**Affiliations:** School of Psychology, Newman Building, University College Dublin, Belfield Campus, Dublin 4, Dublin, Ireland

**Keywords:** Intentions, Partner notification, Screening, Self-efficacy, Sexually transmitted infections

## Abstract

**Background:**

Sexually transmitted infections (STIs) impose burdens on individuals and communities, while their prevalence in young people has risen continually in recent years. Partner notification is an effective public health strategy which can limit STI transmission.

**Aims:**

This study aimed to explore young adults’ sexual health behaviours, attitudes toward STI testing, and feelings toward visiting a sexual health clinic. It also aimed to investigate preferences for partner notification and the role of self-efficacy in people’s intentions to notify a partner for STIs including the human immunodeficiency virus (HIV).

**Methods:**

A quantitative, cross-sectional design was applied. Participants were 400 adults aged 18–34 years (*M* = 23 years; SD = 4.27), recruited from the Republic of Ireland.

**Results:**

Over half of the participants reported never being tested for STIs. These young people placed less importance on undergoing regular STI testing and testing after unprotected sex than their counterparts who had been tested for STIs. Self-efficacy was significantly associated with intentions to notify partner(s) for STIs including HIV.

**Conclusions:**

As STIs are becoming increasingly prevalent in young adults, it is important to gain a deeper understanding of the interventions used to break the transmission chain and how different beliefs and attitudes may affect them. Self-efficacy was a key component in PN intentions, suggesting that the belief in someone’s ability or skillset to perform a sexual health behaviour is positively related to their intention to perform the behaviour.

## Introduction

Sexually transmitted infections (STIs) including the human immunodeficiency virus (HIV) constitute a major public health concern severely affecting maternal, child, and reproductive health with debilitating consequences for societies and economies [1]. Despite the wide range of behavioural and biomedical interventions applied to interrupt the STI transmission chain, the prevalence of STIs remains high worldwide and in Europe, with more than one million people being newly infected with STIs per day [2]. STIs are on the rise in the Republic of Ireland with young people aged 15 to 24 years old accounting for over half of all reported cases [3–5]. For instance, in 2018, young people aged 15 to 24 years old accounted for 49% of all chlamydia cases, 39% of herpes simplex virus cases, and 32% of gonorrhoea cases reported in Ireland [6]. Research conducted among 419 Irish university students found that 90% of respondents were sexually active, with 94% reporting condoms as their most frequent method of contraception. However, many students who were sexually active reported engaging in vaginal (69%), oral (86%), and anal (19%) sex without using a condom in the prior 2 years. Additionally, 44% believed that STIs do not pose a long-term health risk, with 10% of those who were sexually active reporting that they had contracted one or more STIs, most frequently chlamydia [7].

### Partner notification

Partner notification (PN) has been widely applied as a public health strategy to control the spread of STIs. PN is the process of notifying, testing, and, if necessary, treating the sexual partner(s) of the index patient diagnosed with STI(s). When applied successfully, PN helps break the transmission chain, thus decreasing morbidity and mortality rates and subsequently reducing the societal and financial burden of STIs. There are three different approaches of PN defined as (i) patient referral, where the tested patient notifies their sexual partner(s) of a possible STI exposure and refers them to sexual health care services for screening; (ii) provider referral, where the health care professional notifies the sexual partner(s) of the tested patient; and (iii) contract referral, where the health care professional notifies the sexual partner(s) within an agreed time period in the event that the tested patient fails to do so. Research which examined the effectiveness of PN approaches found patient referral to be the most commonly used approach due to individuals’ preferences to notify their own sexual partner(s) themselves [8]. However, the authors also noted that the effectiveness of this PN approach was only 30%, and its contribution toward controlling the spread of STIs was lower than anticipated.

Previous research has found that individuals are more likely to notify their main sexual partner of a possible STI diagnosis than partners who are seen as transmitters or casual or one-time sexual partners before the onset of symptoms [9]. Furthermore, better relationship quality in committed relationships, as measured by stronger emotional and affiliative bonds, has been associated with increased PN, particularly for curable STIs such as syphilis, gonorrhoea, chlamydia, and trichomoniasis [10]. Alam et al. [11] observed that individuals with positive attitudes toward partner referral were more likely to have higher intentions to notify their partners than those with negative attitudes. Furthermore, attitudes toward referring and intentions to notify sexual partners were positively associated with actual PN behaviour. Nuwaha et al. [12] suggested that attitudinal beliefs about PN may influence PN intentions, highlighting that individuals with positive PN attitudes may also conform to positive social norms toward referring a partner. They also suggested that where individuals believe their partner will refuse treatment, they may have lower PN intentions because they have less control over their partner’s subsequent behaviour. However, self-efficacy may affect the implementation of behaviour through increasing persistence in the face of initial failure [12].

### Self-efficacy and sexually transmitted infections

Self-efficacy is defined as a person’s beliefs about their capacity to perform specific behaviours in specific situations [10]. It has been suggested to be a determining factor of present and future health behaviours, in addition to behaviour change. For example, self-efficacy has been reported as an important predictor for behavioural health outcomes such as smoking cessation [13, 14], as well as sexual health behaviour outcomes such as condom use [15]. Self-efficacy is suggested to be a determinant of condom use in young adults, with higher ratings of self-efficacy being associated with a higher likelihood of use. Regarding other health risk behaviours, it has been suggested that smoking-specific self-efficacy may predict smoking cessation intentions [13]. Although there is generally limited research on the role of self-efficacy in sexual health behaviours, there is some evidence identifying self-efficacy as a significant factor in predicting PN behaviour. For example, Schwartz et al. [16] found that higher self-efficacy was associated with more positive attitudes and greater PN intentions for referring sexual partner(s). Furthermore, Fortenberry et al. [10] reported that self-efficacy was a predictor of PN intentions at the 1-month follow-up of STI treatment.

## The present study

The World Health Organisation (WHO) and the Joint United Nations Programme on HIV/acquired immune deficiency syndrome (AIDS) recommend that PN is carried out voluntarily within supportive and permitting legal and social settings [17]. While Ireland adheres to the WHO international guidelines for PN regarding HIV transmission, when managing the spread of STIs, Irish PN recommendations follow the guidelines of the British Association for Sexual Health and HIV [18] and of the Centers for Disease Control and Prevention (CDC) guidelines [19]. This highlights the importance of understanding PN within the context of Ireland, which will help to inform the development of culturally specific and relevant guidelines. This realisation becomes increasingly important considering that in recent decades, Ireland has a seen dramatic rise in the prevalence of STIs [20]. Although PN has become a common and effective practice in the worldwide control of STIs, research in this field is limited in the European region as well as in Ireland [21]. The majority of existing evidence on the role of PN in behaviours related to STIs still focuses almost solely on HIV without including other STIs (e.g. Bull et al. [22]). Hence, the present study aimed to investigate young adults’ sexual health behaviours and attitudes toward STI testing and visiting sexual health clinics. Furthermore, it aimed to explore preferences for partner notification and the role of self-efficacy in intentions to notify a sexual partner for a potential STI infection including HIV and non-HIV STIs.

## Method

### Participants

Participants were 400 young adults aged 18–34 years old (*M* = 23 years; SD = 4.27) recruited as part of a larger study targeting the general population in the Republic of Ireland. Detailed demographic characteristics of the sample are presented in Table 1.

**Table 1:** Demographic characteristics of the sample

**Characteristic**		**Number**	**Percent**
Gender (*n* = 400)	Female	276	69.0%
	Male	116	29.0%
	Do not identify as female or male	8	2.0%
Ethnicity (*n* = 400)	White/White Irish	373	93.3%
	Other ethnicities	27	6.8%
Education (*n* = 395)	Higher secondary	174	44.1%
	Third level—undergraduate	164	41.5%
	Third level—postgraduate	57	14.4%
Sexual orientation (*n* = 398)	Heterosexual	295	74.1%
	Homosexual	34	8.5%
	Bisexual	60	15.1%
	Asexual	9	2.3%
Employment status (*n* = 398)	Employed	148	37.2%
	Self-employed	8	2.0%
	Unemployed	22	5.5%
	Student	220	55.3%
Place of residence (*n* = 400)	Urban (city/town)	337	84.3%
	Rural (countryside)	63	15.8%

## Materials

### Demographic information

Demographic information was collected on age, gender, ethnicity, education, sexual orientation, employment status, and place of residence (urban, rural).

### STI testing, sexual activity, and sexual health behaviours

Questions on sexual activity asked participants whether they were sexually active in the past year (yes, no), whether they were in a committed relationship (not in a committed relationship, in a committed relationship, married/cohabiting), and their total number of sexual partners (no partners, 1 or 2 partner(s), 3–5 partners, 6–9 partners, > 10 partners). Questions about sexual health behaviours included frequency of protection use (condoms, dental dams). Responses ranged from 1–5 (never, rarely, sometimes, most of the time, always). We also asked participants whether they had previously been tested for STIs (yes, no). Finally, we asked participants whether they had received a STI diagnosis in the past (yes, no).

### Attitudes toward STI testing and feelings about visiting a sexual health clinic

Attitudes toward STI testing were measured using two items. Participants were asked about how important they believe (i) STI testing is even without symptoms and (ii) STI testing following unprotected sex. Items were rated on a 5-point Likert scale ranging from 1 (strongly disagree) to 5 (strongly agree). Higher scores indicated higher perceived importance of STI testing in each instance.

Participants were asked to indicate their feelings about visiting a sexual health clinic (uneasy, judged, and comfortable) using three items adapted from Shepherd and Harwood [23]. Items were rated on a 5-point Likert scale ranging from 1 (strongly agree) to 5 (strongly disagree). The three items were reverse scored so that higher scores indicated feeling more uneasy, more judged, and more comfortable when visiting a sexual health clinic.

### Partner notification history, preferences, and intentions

Information on PN history was collected using two items. Participants were asked (i) whether they have ever been notified by a partner and/or a health clinic for potential exposure to STIs and (ii) whether they have ever notified a sexual partner for potential exposure to STIs.

Preferences for PN (i.e. methods and treatments) were measured using two items. Participants were asked to indicate (i) their preferred method of PN from three options (inform partner myself, a health provider informs partner, or would not inform partner) and (ii) which treatment(s) they would prefer to give their partner in case of potential exposure to STIs (arrange a clinic appointment, give them a referral sheet, give them an STI pamphlet, give them antibiotic pills or an antibiotic prescription, or would not do any of the above). For preferred PN treatments, participants were asked to select as many options as applied (i.e. multiple responses could be selected).

Intentions to notify partners specifically for HIV and for non-HIV STIs were measured with two individual items: ‘If you were diagnosed with HIV, how likely would you be to notify your sexual partner’ and ‘If you were diagnosed with a sexually transmitted infection other than HIV (e.g. syphilis, chlamydia, gonorrhoea etc., how likely would you be to notify your sexual partner’. Responses ranged from 1 (very likely) to 5 (very unlikely). Items were reverse scored to make higher scores indicative of greater intentions of notifying a partner.

### Self-efficacy

Self-efficacy was measured using a self-efficacy questionnaire developed by Nuwaha et al. [12]. Participants were asked to rate the likelihood of notifying and referring a partner to a sexual health clinic on 12 different occasions. Each occasion was represented by one of 12 items. For example, ‘I would refer my partner if I had the words to tell him/her’. Responses ranged from 1 (extremely likely) to 7 (extremely unlikely). Items were reversed scored to make higher scores indicative of a greater likelihood to refer the partner on each occasion. The internal consistency of the scale was excellent for the present sample (Cronbach’s *α* = 0.90). For the purposes of the present study, we calculated an individual mean score for each item.

## Procedure

Data for this quantitative cross-sectional study were collected anonymously online from January 2018 through April 2019. Participants were recruited through different channels including social media and through using the snowball technique, while flyers containing the study information were shared online. No paid advertisements were used in the participant recruitment process and no incentives were offered to participants in exchange for taking part. Participants were eligible to take part if they were 18 years old or older; however, the present study used data only from participants aged 18–35 years old. Participants were presented with detailed information about the study and asked to provide their electronic consent before starting to complete the survey. Ethical approval for this project was obtained from the University College Dublin Human Research Ethics Committee-Humanities. The procedures used in this study adhere to the tenets of the Declaration of Helsinki.

## Results

### Sexual activity and sexual health behaviours

Table 2 presents sexual activity and sexual health behaviours reported by young adults in our sample in detail. Approximately 88% reported being sexually active during the past year. Most participants (35.3%) reported having one or two sexual partners during their lifetime, while 22.7% reported having more than 10. As Table 2 shows, 57.9% of participants indicated that they use protection during their sexual encounters always or most of the time, while 16.9% reported that they always engage in unprotected sex. However, out of the 67 participants who reported always having unprotected sex (i.e. never use protection), most (80.6%) were in a committed relationship, while only 6.5% of those who were not in a non-committed relationship reported never using protection during sex.

**Table 2:** Frequencies for sexual activity and sexual health behaviours

		**Number**	**Percent**
Sexually active in the past year (*n* = 398)	Yes	351	88.2%
	No	47	11.8
Relationship status (*n* = 400)	Not in a committed relationship	204	51.0%
	In a committed relationship (includes married/cohabiting)	196	49.0%
Total number of sexual partners (*n* = 397)	1–2	140	35.3%
	3–5	86	21.7%
	6–9	56	14.1%
	> 10	90	22.7%
	Never been sexually active	25	6.3%
Protection use (*n* = 396)	Always	125	31.6%
	Most of the time	104	26.3%
	Sometimes	60	15.2%
	Rarely	40	10.1%
	Never	67	16.9%

### STI testing

Table 3 presents information on prior STI testing for the present sample. Over half of the study participants (54.8%) reported never being tested for one or more STIs such as syphilis, chlamydia, HIV, gonorrhoea, trichomoniasis, human papilloma virus (HPV), herpes simplex virus (HSV), and hepatitis B. Notably, of those participants who had more than two sexual partners in their lifetime, 39.4% reported never being tested for one or more STIs. Almost half of the participants (45.3%) reported that they had previously been tested for one or more STIs. When participants were asked whether they believe they ever had contracted one or more STIs, 7.1% indicated that they had an infection, while 4.5% (*n* = 18) responded that they were unsure. Chlamydia was the most frequently reported STI (*n* = 16, 4%) followed by HPV (*n* = 9, 2.3%). Of the total sample, 6.5% (*n* = 28) reported that they had been diagnosed by a healthcare professional with one or more STIs.

**Table 3:** Frequencies for prior testing for, and diagnosis of, sexually transmitted infections

**Variable**		**Number**	**Percent**
STI testing (*n* = 400)	No	219	54.8%
	Yes	181	45.3%
	Chlamydia	155	38.8%
	HIV	129	32.3%
	Gonorrhoea	129	32.3%
	Hepatitis B	126	31.5%
	Syphilis	114	28.5%
	HSV	98	24.5%
	HPV	92	23.0%
	Trichomoniasis	64	16.0%
STI past diagnosis (*n* = 396)	No	350	88.4%
	Unsure	18	4.5%
	Yes	28	7.1%
	Chlamydia	16	4.0%
	HPV	9	2.3%
	Gonorrhoea	6	1.5%
	HSV	2	0.5%
	HIV/AIDS	2	0.5%
	Hepatitis B	1	0.3%
	Syphilis	1	0.3%

*AIDS *acquired immunodeficiency syndrome, *HIV *human immunodeficiency virus, *HPV *Human papillomavirus, *HSV *herpes simplex virus

### Attitudes toward STI testing and clinic attendance

Regarding attitudes toward STI testing, 55.4% of participants strongly agreed that individuals should get tested even without symptoms, while 67.1% strongly agreed that testing is essential following unprotected sex.

Independent samples *t*-tests were conducted in order to investigate potential differences in attitudes toward STI testing between participants who had been tested for STIs and those who had never been tested (i.e. by prior STI testing status). As can be seen in Table 4, participants who had never been tested for STIs believed it was less important to test for STIs regularly and after unprotected sex than their counterparts who had been tested for one or more STIs. Further, participants who had never been tested for STIs reported feeling more uneasy, more judged, and less comfortable about visiting a sexual health clinic than their counterparts who had been tested for STIs in the past.

**Table 4:** Differences in attitudes toward STI testing (1–5, strongly disagree–strongly agree) and feelings about visiting a sexual health clinic (1–5, higher scores indicative of feeling more uneasy, judged, comfortable), according to prior STI testing status (*n* = 395)

		Prior STI testing—never	Prior STI testing—yes	*t*-test (df)	eta-squared
		Mean (SD)	Mean (SD)		
Attitude toward STI testing	Regular testing important even without symptoms	4.17 (0.89)	4.66 (0.63)	− 6.34* (386)	0.09
	Testing essential following unprotected sex	4.43 (0.85)	4.71 (0.66)	− 3.69* (392)	0.03
Feelings about visiting a sexual health clinic	Feel uneasy visiting a sexual health clinic	3.71 (1.16)	2.89 (1.39)	6.34* (351)	0.09
	Feel judged visiting a sexual health clinic	3.41 (1.28)	2.58 (1.27)	6.43* (398)	0.09
	Feel comfortable visiting a sexual health clinic	2.74 (1.15)	3.47 (1.21)	− 6.16* (398)	0.09

*p < 0.001

### Partner notification

#### Partner notification history and preferences (methods and treatments)

As can be seen in Table 5, 9% of participants reported that they have notified a sexual partner for potential exposure to STIs, and 7% reported that a sexual partner had notified them. Participants were asked what their preferred method of notifying a partner would be in the event they were diagnosed with STI(s) themselves. Most participants reported that they would prefer to notify their partners themselves (88.1%, i.e. patient referral), with only 10.9% reporting that they would prefer a healthcare provider to inform their sexual partner(s) (i.e. provider referral). Only 1% indicated that they would not inform their sexual partners at all. When participants were asked to indicate their preferred method of treatment, the majority (70%) reported that they would prefer to arrange a clinic appointment for their partner(s), followed by those (59.3%) who reported that they would prefer to give a referral sheet to their partner. Notably, 9.5% of participants reported that they would not engage in any of the presented options of notifying their sexual partner(s) (see Table 5 for details).

**Table 5:** Frequencies for partner notification history, and preferences for PN (method and treatment)

**Variable**		**Number**	**Percent**
PN history (*n* = 383)	Notified *by* a partner	27	7.0%
	Notified a partner	35	9.1%
	Notified by a health clinic (*n* = 386)	13	3.3%
Preferred method of PN (*n* = 386)	Inform partner myself (i.e. patient referral)	340	88.1%
	Health care provider informs partner (i.e. provider referral)	42	10.9%
	Would not inform partner	4	1.0%
Preferred treatment of PN (*n* = 400)	Clinic appointment	280	70.0%
	Referral sheet	237	59.3%
	STI pamphlet	232	58.0%
	Antibiotic prescription	174	43.5%
	Antibiotic pills	157	39.3%
	Would not give any of the above	38	9.5%

*PN *partner notification

#### Intentions to notify partner(s)

We asked participants about their intentions to notify a sexual partner in the case that they have been hypothetically diagnosed with HIV and with non-HIV STIs. The majority (89%) of participants reported they would be very likely to inform a sexual partner if they were diagnosed with HIV, while 72.5% reported that they would be very likely to inform a partner if they were diagnosed with non-HIV STIs.

#### Self-efficacy for partner notification

To examine the relationship between self-efficacy for partner notification and intentions to notify partners for HIV and non-HIV STIs, we conducted a series of two-tailed Pearson’s product-moment correlations. Table 6 presents the correlation matrix between the likelihood of notifying a sexual partner on each occasion and intentions to notify a sexual partner separately for HIV and non-HIV STIs. All correlations had a positive direction, and all except one were significant (see Table 6). This indicates that the higher the likelihood to perform the behaviour (i.e. to notify partner(s) for potential exposure to STIs) on each occasion, the greater the intention to notify partner(s) both for HIV and non-HIV STIs. All self-efficacy occasions showed stronger correlations with intentions to notify partners for non-HIV STIs than with intentions to notify partners for a potential HIV infection.

**Table 6:** Correlation matrix for individual occasions of self-efficacy and intentions to notify partner(s) for non-HIV STIs and HIV (*n* = 400)

Self-efficacy	M (SD)	PN intentions for non-HIV STI(s)	PN intentions only for HIV
I would notify my partner…		Pearson’s *r*	
if I had the words to tell her/him	6.48 (0.82)	0.50**	0.30**
if I had a letter from a healthcare provider	6.47 (0.90)	0.39**	0.23**
if she/he was the source of infection	6.75 (0.72)	0.10	0.04
if I were able to discuss STIs with him/her	6.58 (0.80)	0.33**	0.24**
if I knew she/he would get free treatment	6.58 (0.89)	0.35**	0.16**
if I were the source of infection	6.66 (0.80)	0.49**	0.34**
if I felt comfortable talking with her/him about it	6.72 (0.72)	0.33**	0.18**
even if I knew she/he has other men/women	6.35 (1.09)	0.26**	0.23**
even if I will never have sex with her/him again	6.26 (1.15)	0.40**	0.34**
even if I feared that our sexual relationship will be known to others	6.21 (1.10)	0.47**	0.23**
if I was examined in a laboratory	6.14 (1.12)	0.32**	0.16*
if I knew the trick to bring her/him to the clinic	6.01 (1.33)	0.27**	0.12*

*p < 0.05; **p < 0.01

Regarding intentions to notify partners for non-HIV STIs, the strongest correlation was observed with the items, ‘I would notify my partner if I had the words to tell her/him’ (*r* = 0.50, *p* < 0.001) and ‘if I were the source of infection’ (*r* = 0.49, *p* < 0.001), followed by the item, ‘…even if I feared that our sexual relationship will be known to others’ (*r* = 0.47, *p* < 0.001; see Table 6). The weaker relationships between intentions to notify partners for non-HIV STIs were observed with the item, ‘if I knew the trick to bring her/him to the clinic’ (*r* = 0.27, *p* < 0.001) and the item ‘…if I knew she/he has other men/women’ (*r* = 0.26, *p* < 0.001).

Regarding intentions to notify partners to get tested for HIV, the strongest correlation was observed with the item, ‘I would notify my partner if I were the source of infection’ (*r* = 0.34, *p* < 0.001) followed by the item ‘I would notify my partner even if I would never have sex with her/him again’ (*r* = 0.34, *p* < 0.001). The weaker relationships between intentions to notify partners for HIV were observed with the items ‘I would notify my partner if I was examined in a laboratory’ and ‘…if I knew she/he would get free treatment’ (*r* = 0.16, *p* < 0.001) followed by the item ‘…if I knew the trick to bring her/him to the clinic’ (*r* = 0.12, *p* = 0.15). Only one item, ‘I would notify my partner if she/he were the source of infection’, was not significantly associated with intentions to notify a partner for a potential HIV infection (*r* = 0.04, *p* = 0.4).

## Discussion

The present paper reported findings on young adults’ sexual activity and sexual health behaviours, attitudes toward STI testing and visiting a sexual health clinic, and preferences for PN, as well as on the role of self-efficacy in intentions to notify a partner for a potential STI diagnosis including HIV.

### Attitudes toward STI testing and clinic attendance

Over half of our sample (54.8%) reported never being tested for STIs. Of those, 39.4% reported having more than two sexual partners in their lifetime. Low testing rates among young adults have been also reported by other studies from different countries. For instance, a US study in a large cohort of young adults reported that from 2013–2019, 47% of women and 78% of men in their sample had not received an STI test [24]. A UK study of young people aged 16–24 years old found that 52% of participants had no prior experience of STI testing [25], while similar findings were reported in a cohort of young adults in Canada (64% had never been tested) [26]. Low testing rates become increasingly important and concerning when taking into consideration that in recent years in the Republic of Ireland, the young adult age group (up to 35 years old) has consistently had the highest age-specific incidence rates of STI infections [27, 28]. Thus, the prevalence of STIs may be even higher than is reported among Irish youth considering the low STI testing rates in this age cohort.

Attitudes toward STI testing and feelings about visiting a sexual health clinic differed between young adults who had previously been tested for STIs and their counterparts who had never been tested. Participants who had been tested previously considered it more important to undergo regular STI testing even without symptoms than those who had never been tested. The moderate to large effect size (*η*^*2*^ = 0.09) indicates that this difference in attitude is substantial between the two groups, which may explain the STI screening practices. Similarly, young adults who have never been tested did not deem testing as essential following unprotected sex, in comparison to their counterparts who had been tested previously. However, the small effect size (*η*^*2*^ = 0.03) shows that this difference may not be substantial. A qualitative study in the USA reported that almost half of their sample (adult men who have sex with men (MSM) and transgender women) were not concerned about syphilis infection, and some participants mentioned that a lack of previous infection contributed to their lack of concern [29]. Certainly, according to the Theory of Planned Behaviour [30] an individual’s behaviour, of which intention is a direct antecedent, depends on their behavioural beliefs and attitudes. Hence, our finding that participants with no experience of STI testing do not consider it as important to undergo regular testing as those with experience of STI testing becomes less surprising. Evidence supporting this has been reported in the literature, for example a systematic review reported that people with previous knowledge of STIs and testing were more likely to accept STI testing [31]. However, one study in the review reported that negative experiences with previous STI testing discouraged further testing [32], making the nature of the testing experience and the training of the healthcare professionals involved even more important.

Young adults from our sample who had never been tested for STIs reported feeling significantly more uneasy and judged and less comfortable in relation to visiting a sexual health clinic than their counterparts who had been tested. The moderate to large effect sizes (*η*^*2*^ = 0.09 for each) indicate that these differences are substantial. This corroborates existing evidence from a recent systematic review indicating that young people would seek STI screening from testers with non-judgmental attitudes [31]. It is also in line with research indicating that young adults who report unfavourable attitudes toward visiting a sexual health clinic are less likely to get tested for STIs [23]. Decisions to undergo STI testing can be influenced by factors such as people’s health beliefs, knowledge, previous experience, access to services, attitudes, and perceptions of social or peer norms [25, 33–36]. Negative self-conscious emotions like guilt, shame, and embarrassment are also common when seeking STI care. Difficulty managing these emotions can lead people to drop out of the STI testing process [36]. Experiencing these emotions can often mean that people do not notify their partner(s) of their STI-positive status, thus increasing transmission rates and risks of STI-related sequalae [37, 38].

### Partner notification

Most participants reported that they would be very likely to notify their sexual partners for a HIV/STI diagnosis. However, those who indicated that they would refer their partner for HIV (89%) were higher than those who would refer their partner for non-HIV STIs (72.5%). Although in both cases the likelihood of notifying partners is high, this indicates that when it comes to HIV, people appear to be more driven to proceed with PN. This may be due to HIV being perceived as a life-threatening infection with severe consequences if not treated properly [39]. A study of HIV-negative MSM found that 77% of the sample perceived HIV as serious, and negative social consequences contributed to this perception [40]. Further, perceiving HIV as *not* serious was associated with increased sexual risk behaviours. The difference we observed in PN intentions for HIV versus non-HIV STIs could indicate that our sample does not perceive non-HIV STIs as threatening, perhaps because they are curable, and therefore, there are fewer perceived social or psychological implications attached to them. However, non-HIV STIs can have severe and long-term consequences if left untreated. For example, untreated chlamydial infection can lead to infertility, ectopic pregnancy, and chronic pelvic pain [41]. This is especially concerning given that we found chlamydia was the most commonly diagnosed STI in our sample.

Most participants (88.1%) indicated that they would prefer to notify their sexual partners themselves for potential exposure to STIs (patient referral), with only 10.9% indicating that they would prefer a healthcare provider to perform this action (provider referral). This contradicts a recent study of university students from South Africa where over half (59%) of young adults reported that they would prefer a doctor to notify their partners in the event of an STI [42]. However, a study from Botswana showed that most young adult patients diagnosed with an STI notified their partners themselves and indicated that this would also be their future preference for partner notification [43]. Taken together, these suggest that PN is potentially influenced by the cultural context as well as the health system embedded in that context, thus any decisions to inform policy on PN practices should be considered within each country’s unique cultural and health system context.

### Self-efficacy

Our findings highlight the role of self-efficacy in intentions to notify partner(s) for STIs and extend previous research [44] by identifying specific aspects of self-efficacy that could motivate individuals to engage in PN. Most hypothetical occasions related to self-efficacy showed significant, positive correlations with intentions to notify partner(s) for HIV/STIs. This suggests that, indeed, the belief in someone’s ability or skillset to perform sexual health behaviour is positively related to their intention to perform the behaviour. This is in line with findings from Gursahaney et al. [45], who reported that stronger self-efficacy related to PN was linked to an increased likelihood to perform the behaviour. The role of self-efficacy in PN intentions can be further substantiated by Nuwaha et al. [12] who found that women who believed their partner would refuse STI treatment were less likely to notify them about an STI. People may be less likely to notify a partner as they perceive less control over the situation which follows (i.e. whether their partner seeks treatment or not), thus linking into the concept of self-efficacy itself (i.e. a person’s sense of control over their environment and behaviour).

Participants’ intentions to notify a partner for STIs/HIV were stronger for an occasion where participants *themselves* were the source of an infection than it was for when *their partner* was the source. This may indicate that participants could feel a personal burden or sense of guilt if they were to be the source of an STI. On occasions where participants *themselves* were the source of infection, PN intentions for non-HIV STIs were stronger than intentions for HIV. This could reflect HIV-related stigma in our sample, as well as the anticipation of social or psychological consequences associated with HIV [40]. We saw an insignificant relationship between PN intentions for HIV and the self-efficacy occasion wherein *the partner* is the infection source. This aligns with the findings of Mathews et al. [9], who reported that individuals were less likely to notify sexual partners who were viewed as transmitters. However, it is possible that on this occasion, participants could believe their partner is already aware of the potential infection and thus do not need to be notified. Alternatively, this could also reflect resentment or anger toward a partner if they are viewed as a transmitter of HIV, even more so than of a non-HIV STI which may be widely perceived as less serious. HIV stigma and discrimination is not a new discovery [e.g. 46–48], but our findings suggest that these are still salient in Ireland.

## Limitations

This study has some limitations that should be considered when interpreting results. First, the majority of participants were females in committed heterosexual relationships, with an undergraduate/postgraduate education attainment and living in urban areas. Thus, caution is needed when generalising findings in groups with different demographic characteristics. Second, this study applied a cross-sectional design, therefore making it impossible to monitor changes in PN intentions over time. Future studies should employ longitudinal measurements to test the stability of intentions over time, especially comparing those in a committed and in non-committed relationship. Finally, we did not ask participants about their preferred methods of PN by a partner, as this was beyond the scope of the present study. This would offer a more holistic investigation on PN preferences which can be addressed by future research.

## Implications for research and practice

Our findings highlight the low STI screening rate in young adults in the Republic of Ireland with over half of our sample reporting never being tested. The significant differences in attitudes toward STI testing and in feelings of being judged in relation to visiting a sexual health clinic between people who have and have not been tested indicate that further action needs to be taken to address the stigma and shame associated with STI screening and sexual health clinic attendance which persists in Irish society. In previous years, there have been numerous sexual health awareness events and campaigns in Ireland, which may have increased awareness of sexual health and STIs in the general population. The concept of sex-positivity, involving healthy attitudes, positive relationships, education, and safety, is being increasingly acknowledged in many cultures, including Ireland, which may also have contributed to better awareness of sexual health matters. Future campaigns could focus on reducing the stigma associated with STI testing and attending sexual health clinics in young adult populations. Further, offering training to health professionals who carry out STI tests or work in sexual health clinics could help to foster positive testing environments and ensure that patients perceive the testing procedures positively and subsequently continue to test regularly in the future.

Evidence suggests that the provision of tailored STI screening services is pivotal in facilitating access by young people with diverse sexual orientations and ethnic backgrounds [31]. Future research should explicitly investigate attitudes, as well as the needs of subgroups of young adults from the lesbian, gay, bisexual, and transgender community in the Republic of Ireland. Different ethnic groups of young adults should be also targeted. This will generate valuable evidence that can help inform policy decisions regarding the provision of sexual health services in a more holistic and inclusive manner accounting for sexual and ethnic diversity. PN is an important secondary method of preventing the spread of STIs. Effective PN requires a better, deeper understanding of young people’s preferences for PN methods within the Irish context, as findings suggest that cross-cultural differences may exist. Research could investigate preferences for a number of specific and feasible PN methods within Ireland, such as contacting partners by letter, PN slip, email, phone, etc. Moreover, this should investigate preferences across sexual orientation, gender, and ethnic group. Finally, the role of self-efficacy in clinical programmes and educational campaigns has been somewhat overlooked, but our findings suggest that this is an important aspect to consider. Strategies for boosting self-efficacy for PN could involve helping people manage and control their own sexual health behaviours and stop STI/HIV transmission going forward. Campaigns could focus on changing the narrative from what *they/others* have done (i.e. the sexual partner transmitting an infection), to what *you* personally can do to stop the transmission chain. An effective example of this narrative from the recent COVID-19 pandemic involved an animated video, wherein a row of matches are shown catching on fire until one steps aside and stops the blaze [49]. This artistic campaign promoted social distancing to prevent COVID-19 spread and aimed to get the message across to young people in particular.

## Conclusions

As STIs are becoming increasingly prevalent in young adults, it is important to gain a deeper understanding of the interventions used to break the transmission chain and how different beliefs and attitudes may affect them. By exploring PN intentions among young Irish adults, this study showed that participants were more likely to notify their sexual partner(s) when it came to HIV as opposed to other non-HIV STIs while preferring to notify them personally. This highlights participants’ awareness of the severity of HIV (i.e. life-threatening) and suggests an act of accountability for their actions in exposing the health of another person to potential risk. Furthermore, self-efficacy was a key component in PN. This suggests that the belief in someone’s ability or skillset to perform sexual health behaviour is positively related to their intention to perform the behaviour. Despite the important role self-efficacy appears to play in PN intentions, relatively few efforts have been made to address and account for this factor in clinical intervention and educational programmes, particularly in Ireland. Findings suggest that there is a need to educate the public about self-efficacy and PN, regardless of whether the diagnosis is for HIV or another non-HIV STI. This could be implemented through incorporating information regarding the beneficial outcomes of self-efficacy on PN practices and the consequent benefit of effective PN, into sexual health awareness campaigns. Our findings provide important information on the role of self-efficacy in intentions to notify a partner; however, further research is needed to gain a better understanding across different population groups and inform future educational campaigns.

## References

[1] World Health Organisation (2018) Report on global sexually transmitted infection surveillance, 2018. Switzerland. https://www.who.int/publications/i/item/9789241565691

[2] World Health Organisation (2020) Sexually transmitted infections (STIs) key facts.

[3] Cronin M, Domegan L (2003) Incidence of STIs continues to rise in the Republic of Ireland. Weekly releases (1997–2007) 7:2310. 10.2807/esw.07.42.02310-en 10.2807/esw.07.42.02310-en

[4] Irvine N, Doherty L (2005) Sexually transmitted infections on the rise in Northern Ireland. Weekly releases (1997–2007) 10:2636. 10.2807/esw.10.05.02636-en 10.2807/esw.10.05.02636-en 16685104

[5] Satterwhite CL, Torrone E, Meites E et al (2013) Sexually transmitted infections among US women and men: prevalence and incidence estimates, 2008. Sex Transm Dis 40:187–193. 10.1097/OLQ.0b013e318286bb53 23403598 10.1097/OLQ.0b013e318286bb53

[6] Health Protection Surveillance Centre (2019) Sexually transmitted infections (STIs) among young people in Ireland, 2018. Dublin

[7] Lally K, Nathan VY, Dunne S et al (2015) Awareness of sexually transmitted infection and protection methods among university students in Ireland. Ir J Med Sci 184:135–142. 10.1007/s11845-014-1073-8 24510451 10.1007/s11845-014-1073-8

[8] Bjekić M, Vlajinac HD (2017) Partner notification for gonorrhea and syphilis in Belgrade. Serb. J. Dermatol. Venerol 9:43–48 10.1515/sjdv-2017-0006

[9] Mathews C, Kalichman MO, Laubscher R et al (2018) Sexual relationships, intimate partner violence and STI partner notification in Cape Town, South Africa: an observational study. Sex Transm Infect 94:144. 10.1136/sextrans-2017-053434 29191815 10.1136/sextrans-2017-053434 PMC5870461

[10] Fortenberry JD, Brizendine EJ, Katz BP, Orr DP (2002) The role of self-efficacy and relationship quality in partner notification by adolescents with sexually transmitted infections. Arch Pediatr Adolesc Med 156:1133–1137. 10.1001/archpedi.156.11.1133 12413343 10.1001/archpedi.156.11.1133

[11] Alam N, Streatfield PK, Khan SI et al (2010) Factors associated with partner referral among patients with sexually transmitted infections in Bangladesh. Soc Sci Med 71:1921–1926. 10.1016/j.socscimed.2010.09.009 20943297 10.1016/j.socscimed.2010.09.009 PMC2991073

[12] Nuwaha F, Faxelid E, Wabwire-Mangen F et al (2001) Psycho-social determinants for sexual partner referral in Uganda: quantitative results. Soc Sci Med 53:1287–1301. 10.1016/s0277-9536(00)00410-x 11676401 10.1016/s0277-9536(00)00410-x

[13] Leganger A, Kraft P, R⊘ysamb E (2000) Perceived self-efficacy in health behaviour research: conceptualisation, measurement and correlates. Psychol Health 15:51–69. 10.1080/08870440008400288 10.1080/08870440008400288

[14] Tseng MF, Huang CC, Tsai SC et al (2022) Promotion of smoking cessation using the transtheoretical model: short-term and long-term effectiveness for workers in coastal central Taiwan. Tob Use Insights 15:1179173X221104410. 10.1177/1179173X221104410 35677388 10.1177/1179173X221104410 PMC9168925

[15] O’Leary A, Jemmott LS, Jemmott JB (2008) Mediation analysis of an effective sexual risk-reduction intervention for women: the importance of self-efficacy. Health Psychol 27:S180-184. 10.1037/0278-6133.27.2(Suppl.).S180 18377160 10.1037/0278-6133.27.2(Suppl.).S180

[16] Schwartz RM, Malka ES, Augenbraun M et al (2006) Predictors of partner notification for C. trachomatis and N. gonorrhoeae: an examination of social cognitive and psychological factors. J Urban Health 83:1095–1104. 10.1007/s11524-006-9087-9 16817010 10.1007/s11524-006-9087-9 PMC3261298

[17] European Centre for Disease Prevention and Control (2013) Public health benefits of partner notification for sexually transmitted infections and HIV. ECDC, Stockholm. 10.2900/85700 10.2900/85700

[18] McClean H, Radcliffe K, Sullivan A, Ahmed-Jushuf I (2013) 2012 BASHH statement on partner notification for sexually transmissible infections. Int J STD AIDS 24:253–261. 10.1177/0956462412472804 23970656 10.1177/0956462412472804

[19] Centers for Disease Control and Prevention (2021) Partner services for HIV and STDs: a guide for health care providers. Atlanta, Georgia, United States

[20] Davoren MP, Hayes K, Horgan M, Shiely F (2014) Sexually transmitted infection incidence among adolescents in Ireland. J Fam Plann Reprod Health Care 40:276–282. 10.1136/jfprhc-2013-100596 24916479 10.1136/jfprhc-2013-100596 PMC4174011

[21] Arthur G, Lowndes CM, Blackham J, Fenton KA (2005) Divergent approaches to partner notification for sexually transmitted infections across the European Union. Sex Transm Dis 32:734–741. 10.1097/01.olq.0000175376.62297.73 16314769 10.1097/01.olq.0000175376.62297.73

[22] Bull L, Apea V, Wiggins H et al (2021) BASHH 2018 UK national audit of HIV partner notification. Int J STD AIDS 32:872–877. 10.1177/0956462421990281 33866870 10.1177/0956462421990281

[23] Shepherd L, Harwood H (2017) The role of STI-related attitudes on screening attendance in young adults. Psychol Health Med 22:753–758. 10.1080/13548506.2016.1234715 27636824 10.1080/13548506.2016.1234715

[24] Pleasure ZH, Lindberg LD, Mueller J, Frost JJ (2022) Patterns in receipt and source of STI testing among young people in the United States, 2013–2019. J Adolesc Health 71:642–645. 10.1016/j.jadohealth.2022.04.014 35691850 10.1016/j.jadohealth.2022.04.014

[25] Booth AR, Harris PR, Goyder E, Norman P (2013) Beliefs about chlamydia testing amongst young people living in relatively deprived areas. J Public Health (Oxf) 35:213–222. 10.1093/pubmed/fds082 23042979 10.1093/pubmed/fds082

[26] Cragg A, Steenbeek A, Asbridge M et al (2016) Sexually transmitted infection testing among heterosexual Maritime Canadian university students engaging in different levels of sexual risk taking. Can J Public Health 107:e149–e154. 10.17269/cjph.107.5036 10.17269/cjph.107.5036 PMC6972136 27526211

[27] Health Protection Surveillance Centre (2022) Sexually transmitted infections (STIs) in Ireland: trends to the end of 2021. Health Service Exective, Dublin

[28] Health Protection Surveillance Centre (2021) Sexually transmitted infections (STIs) in Ireland: trends to the end of 2020. Health Service Exective, Dublin

[29] Balán IC, Lopez-Rios J, Dolezal C et al (2019) Low sexually transmissible infection knowledge, risk perception and concern about infection among men who have sex with men and transgender women at high risk of infection. Sex Health 16:580–586. 10.1071/sh18238 31699208 10.1071/sh18238 PMC7725125

[30] Ajzen I (1991) The theory of planned behavior. Organ Behav Hum Decis Process 50:179–211 10.1016/0749-5978(91)90020-T

[31] Gan J, Kularadhan V, Chow EPF et al (2021) What do young people in high-income countries want from STI testing services? A systematic review. Sex Transm Infect 97:574–583. 10.1136/sextrans-2021-055044 34193529 10.1136/sextrans-2021-055044

[32] Kowalczyk Mullins TL, Braverman PK, Dorn LD et al (2010) Adolescent preferences for human immunodeficiency virus testing methods and impact of rapid tests on receipt of results. J Adolesc Health 46:162–168. 10.1016/j.jadohealth.2009.06.015 20113922 10.1016/j.jadohealth.2009.06.015

[33] Balfe M, Brugha R, O’Connell E et al (2010) Where do young Irish women want chlamydia-screening services to be set up? A qualitative study employing Goffman’s impression management framework. Health Place 16:16–24. 10.1016/j.healthplace.2009.07.003 19744875 10.1016/j.healthplace.2009.07.003

[34] Balfe M, Brugha R (2010) Disclosure of STI testing activities by young adults: the influence of emotions and social networks. Sociol Health Illn 32:1041–1058. 10.1111/j.1467-9566.2010.01281.x 20937054 10.1111/j.1467-9566.2010.01281.x

[35] Lorimer K, Reid ME, Hart GJ (2009) “It has to speak to people’s everyday life”: qualitative study of men and women’s willingness to participate in a non-medical approach to Chlamydia trachomatis screening. Sex Transm Infect 85:201–205. 10.1136/sti.2008.031138 19106148 10.1136/sti.2008.031138

[36] Dixon-Woods M, Stokes T, Young B et al (2001) Choosing and using services for sexual health: a qualitative study of women’s views. Sex Transm Infect 77:335–339 11588278 10.1136/sti.77.5.335 PMC1744361

[37] Royer HR, Zahner SJ (2009) Providers’ experiences with young people’s cognitive representations and emotions related to the prevention and treatment of sexually transmitted infections. Public Health Nurs 26:161–172 19261155 10.1111/j.1525-1446.2009.00767.x

[38] McCaffery K, Waller J, Nazroo J, Wardle J (2006) Social and psychological impact of HPV testing in cervical screening: a qualitative study. Sex Transm Infect 82:169–174 16581749 10.1136/sti.2005.016436 PMC2564695

[39] Foreman M, Ní Rathaille N (2016) Not just another long term chronic illness — social work and HIV in Ireland. Practice 28:97–114. 10.1080/09503153.2015.1087494 10.1080/09503153.2015.1087494

[40] Zimmermann HM, van Bilsen WP, Boyd A et al (2021) Prevention challenges with current perceptions of HIV burden among HIV-negative and never-tested men who have sex with men in the Netherlands: a mixed-methods study. J Int AIDS Soc 24:e25715 34449130 10.1002/jia2.25715 PMC8395388

[41] O'Connell CM, Ferone ME (2016) Chlamydia trachomatis genital infections. Microb Cell 3:390–403. 10.15698/mic2016.09.525 10.15698/mic2016.09.525 PMC5354567 28357377

[42] Mokgatle MM, Madiba S, Cele L (2021) A comparative analysis of risky sexual behaviors, self-reported sexually transmitted infections, knowledge of symptoms and partner notification practices among male and female university students in Pretoria, South Africa. Int J Environ Res Public Health. 10.3390/ijerph18115660 34070603 10.3390/ijerph18115660 PMC8198344

[43] Hansman E, Wynn A, Moshashane N et al (2021) Experiences and preferences with sexually transmitted infection care and partner notification in Gaborone, Botswana. Int J STD AIDS 32:1250–1256. 10.1177/09564624211033231 34304619 10.1177/09564624211033231

[44] Niland R, Nearchou F (2020) Self-efficacy and outcome beliefs as predictors of intentions to notify partners for sexually transmitted infections in Ireland. Int J Health Promot Educ 58:42–50. 10.1080/14635240.2019.1671890 10.1080/14635240.2019.1671890

[45] Gursahaney PR, Jeong K, Dixon BW, Wiesenfeld HC (2011) Partner notification of sexually transmitted diseases: practices and preferences. Sex Transm Dis 38:821–827. 10.1097/OLQ.0b013e31821c390b 21844737 10.1097/OLQ.0b013e31821c390b

[46] Fauk NK, Hawke K, Mwanri L, Ward PR (2021) Stigma and discrimination towards people living with HIV in the context of families, communities, and healthcare settings: a qualitative study in Indonesia. Int J Environ Res Public Health. 10.3390/ijerph18105424 34069471 10.3390/ijerph18105424 PMC8159085

[47] dos Santos MML, Kruger P, Mellors SE et al (2014) An exploratory survey measuring stigma and discrimination experienced by people living with HIV/AIDS in South Africa: the people living with HIV Stigma Index. BMC Public Health 14:80. 10.1186/1471-2458-14-80 24461042 10.1186/1471-2458-14-80 PMC3909177

[48] Nachega JB, Morroni C, Zuniga JM et al (2012) HIV-related stigma, isolation, discrimination, and serostatus disclosure: a global survey of 2035 HIV-infected adults. J Int Assoc Physicians AIDS Care 11:172–178 10.1177/1545109712436723 22431893

[49] Izaguirre V, Delcan J (2020) Safety match. Los Angeles

